# High-density lipoprotein-cholesterol levels and risk of cancer in HIV-infected subjects: Data from the ICONA Foundation Cohort

**DOI:** 10.1097/MD.0000000000004434

**Published:** 2016-09-09

**Authors:** Nicola Squillace, Laura Galli, Alessandra Bandera, Antonella Castagna, Giordano Madeddu, Pietro Caramello, Andrea Antinori, Annamaria Cattelan, Franco Maggiolo, Antonella Cingolani, Andrea Gori, Antonella d’Arminio Monforte

**Affiliations:** a Infectious Diseases Clinic, San Gerardo Hospital, University of Milano-Bicocca, Monza; b Infectious Diseases Department, San Raffaele Scientific Institute, Università Vita-Salute San Raffaele, Milan; c Unit of Infectious Diseases, Department of Clinical and Experimental Medicine, University of Sassari, Sassari; d Infectious and Tropical Diseases Unit I, Department of Infectious Diseases, Amedeo di Savoia Hospital, Torino; e Clinical Department, National Institute of Infectious Diseases ‘L.Spallanzani’, Rome; f Division of Infectious Diseases, Padova Hospital, Padova; g Division of Infectious Diseases, Azienda Ospedaliera Papa Giovanni XXIII, Bergamo; h Department of Publich Health, Infectious Diseases, Catholic University, Rome; i Department of Health Sciences, Clinic of Infectious Diseases, ‘San Paolo’ Hospital, University of Milan, Milan, Italy.

**Keywords:** AIDS-defining malignancies, cancer, HDL, HIV, non-AIDS-defining malignancies

## Abstract

Investigation of the relationship between high-density lipoprotein-cholesterol (HDL-c) and the risk of developing cancer in a prospective cohort of human immunodeficiency virus (HIV)-infected patients.

The Italian Cohort of Antiretroviral-naïve Patients Foundation Cohort is an Italian multicenter observational study recruiting HIV-positive patients while still antiretroviral treatment-naïve, regardless of the reason since 1997.

Patients with at least 1 HDL-c value per year since enrollment and one such value before antiretroviral treatment initiation were included. HDL-c values were categorized as either low (<39 mg/dL in males or <49 mg/dL in females) or normal. Cancer diagnoses were classified as AIDS-defining malignancies (ADMs) or non-AIDS-defining malignancies (NADMs). Kaplan–Meier curves and Cox proportional-hazards regression models were used.

Among 4897 patients (13,440 person-years of follow-up [PYFU]), 104 diagnoses of cancer were observed (56 ADMs, 48 NADMs) for an overall incidence rate of 7.7 (95% confidence interval [CI] 6.3–9.2) per 1000 PYFU.

Low HDL-c values at enrollment were associated with higher risk both of cancer (crude hazard ratio [HR] 1.72, 95% CI 1.16–2.56, *P* = 0.007) and of NADM (crude HR 2.50, 95% CI 1.35–4.76, *P* = 0.003). Multivariate analysis showed that the risk of cancer diagnosis was higher in patients with low HDL-c values (adjusted HR [AHR] 1.87, 95% CI 1.18–2.95, *P* = 0.007) in older patients, those patients more recently enrolled, and in those with low current cluster of differentiation 4+ levels, and/or high current HIV-ribonucleic acid.

The multivariate model confirmed an association between HDL-c (AHR 2.61, 95% CI 1.40–4.89, *P* = 0.003) and risk of NADM.

Low HDL-c is an independent predictor of cancer in HIV-1-infected subjects.

## 1 Introduction

Cancer is one of the most important causes of mortality in human immunodeficiency virus (HIV)-infected patients, occurring both as acquired immune deficiency syndrome (AIDS)-defining malignancies (ADMs) and non-AIDS-defining malignancies (NADMs).^[1,2]^

Inflammation is a crucial pathogenetic mechanism involved in cancer development,^[3]^ and inflammation and immune activation are highly prevalent in HIV-infected subjects despite the use of antiretroviral treatment (ART).^[4,5]^

Lipid profile is profoundly influenced by proinflammatory state, which alters predominantly high-density lipoprotein-cholesterol (HDL-c) composition and function.^[6,7]^ Indeed, low HDL-c has been associated with high levels of tumor necrosis factor-alfa (TNF-α)^[8]^ and predominance of a proinflammatory phenotype in monocyte-derived macrophages, suggesting that inflammation could be a risk factor for low HDL-c.^[9]^

Of late, low HDL-c values have been associated with cancer diagnosis.^[10,11]^ Meanwhile, apolipoprotein A1 (ApoA1), an important component of HDL-c, has been demonstrated to have a direct suppressive effect on tumor cells of melanoma in vitro and in vivo.^[12]^ This predominant role of ApoA1 was confirmed by studies that used ApoA1 HDL-c-mimetics peptides to inhibit ovarian and colon cancer development in mouse models.^[13,14]^

Little is known about the predictive value of HDL-c in cancer development in the setting of HIV infection.

HIV affects the ATP-binding cassette transporter A1 (ABCA-1)-dependent cholesterol efflux through Nef protein, reducing ApoA1 and, consequently, lowering HDL-c serum level.^[15]^

Baker et al^[16]^ demonstrated that both HDL-c and ApoA1 increase after the start of ART introduction, at levels which correspond to the degree of inflammation present at entry, suggesting that activation of inflammation pathways contribute to HIV-associated changes in HDL-c.

Cancer risk has also been shown to undergo changes during ART depending on cluster of differentiation 4 (CD4) cell count and HIV viral load (VL) modification.

To shed light on the processes described above, the primary aim of our study was to evaluate the association between HDL-c levels and the development of ADM and NADM in a large cohort of HIV-infected patients initiating ART in Italy.

## 2 Methods

### 2.1 Study design and participants

This is a cohort study of all the HIV-1-infected subjects enrolled in the Italian Cohort of Antiretroviral-Naïve Patients (ICONA) Foundation Cohort Study with at least 1 HDL-c value per year available since enrollment and 1 such value before ART initiation. As annual monitoring of HDL-c is a more recent practice, only subjects enrolled in the cohort since January 2009 were eligible for this study.

The ICONA Foundation Cohort is a cohort of HIV-infected patients which superseded the original ICONA study (see detailed description of this cohort elsewhere),^[17]^ recruiting HIV-positive patients while still ART-naïve, regardless of the reason. On average, CD4 cell counts, HIV VL and other laboratory parameters are measured, and clinical and therapeutical data are collected every 4 months.

Incident cancer cases diagnosed after enrollment were considered in the analyses, focusing on the earliest cancer diagnosis and ignoring subsequent diagnoses in the same patient. Prevalent cases, that is, patients with a cancer diagnosis before enrollment in the ICONA Foundation Cohort, were excluded from the analyses.

Malignancies were classified as ADM or NADM; ADM included Kaposi sarcoma (KS), non-Hodgkin lymphoma, primary central nervous system lymphoma, and invasive cervical cancer; NADM included Hodgkin lymphoma, hepatocellular carcinoma (HCC), lung cancer, larynx cancer, anal cancer, stomach cancer, colon cancer, rectal cancer, skin cancer, melanoma, breast cancer, prostate cancer, testicular cancer, bladder cancer, pancreas cancer, and renal cancer.

Patients were followed up from enrollment date to earliest cancer diagnosis, latest clinical visit, or lost-to-follow-up or death. Data freezing for this analysis was June 2015.

### 2.2 Statistical analysis

Results were described as median (interquartile range [IQR]) or frequency (%), unless otherwise specified.

High-density lipoprotein-cholesterol was used either as a continuous variable or as a categorical variable in one of the 2 classes: low (<39 mg/dL in males or <49 mg/dL in females) or normal. Our analysis factored in not only the last HDL-c value taken before cancer diagnosis, but each HDL-c value collected over the entire study period.

Crude rates of incident cancer (incidence rate [IR]) were calculated as the number of cancer diagnoses divided by the number of person-years of follow-up (PYFU), and were expressed per 1000 PYFU; confidence intervals (CIs) for the rates were calculated assuming a Poisson distribution.

The Kaplan–Meier method was used to estimate the probability of cancer occurrence based on HDL-c level at enrollment; curves were compared by use of the log-rank test.

Three multivariate Cox proportional-hazards regression models were used to evaluate the association between HDL-c and the risk of cancer, and the risk of specific cancer categories (ADM and NADM), adjusting for a number of potential confounders.

At univariate analysis, we determined if HDL-c was better modeled as a continuous variable or as a categorical variable (low vs normal), based on the Akaike information criterion (AIC). As the estimated AICs were very similar (1–2 points AIC difference, with no clear evidence of one model's superiority), HDL-c was considered to be a categorical variable in the 3 multivariate models.

Factors included in the multivariate models were either fixed (age, sex, smoke, hepatitis C virus-antibody [HCV-Ab], hepatitis B surface antigen [HBsAg], lowest ever [nadir] CD4, calendar year of enrollment) or time-updated variables (use of ART, current CD4, current HIV-ribonucleic acid [HIV-RNA] [on the log10 scale], current HDL-c, and current triglycerides).

Missing values of categorical variables were grouped into specific categories, with no loss of observations at multivariate analysis. Missing values of continuous variables, on the other hand, led to a loss of observations, at which point the number of events (on average 88%) effectively retained in each multivariate model was indicated.

The multivariate models were refit after additional testing of current CD4/cluster of differentiation 8 (CD8) ratio, a known predictive factor of non-AIDS-related events.^[18]^ The multivariate analyses were also restricted to that subgroup of patients exposed to ART, and inclusion in the analyses of cancer was restricted to those cancer diagnoses which were performed subsequent to ART initiation. Finally, multivariate analyses were recalculated after exclusion of cancer diagnoses recorded within the first 6 months since enrollment, to exclude those diagnoses which might be considered prevalent, rather than incident events.

All reported *P* values were 2-sided and considered to be statistically significant if below 0.05. The analyses were performed using statistical analysis system (SAS) Software, release 9.2 (SAS Institute, Cary, NC).

### 2.3 Ethics

All patients signed consent forms to participate in the ICONA Foundation Study, in accordance with the ethics standards of the committee on human experimentation and the Helsinki Declaration (1983 revision).

## 3 Results

In all, 4897 patients participated. Demographics and clinical characteristics are shown in Table 1.

**Table 1:**
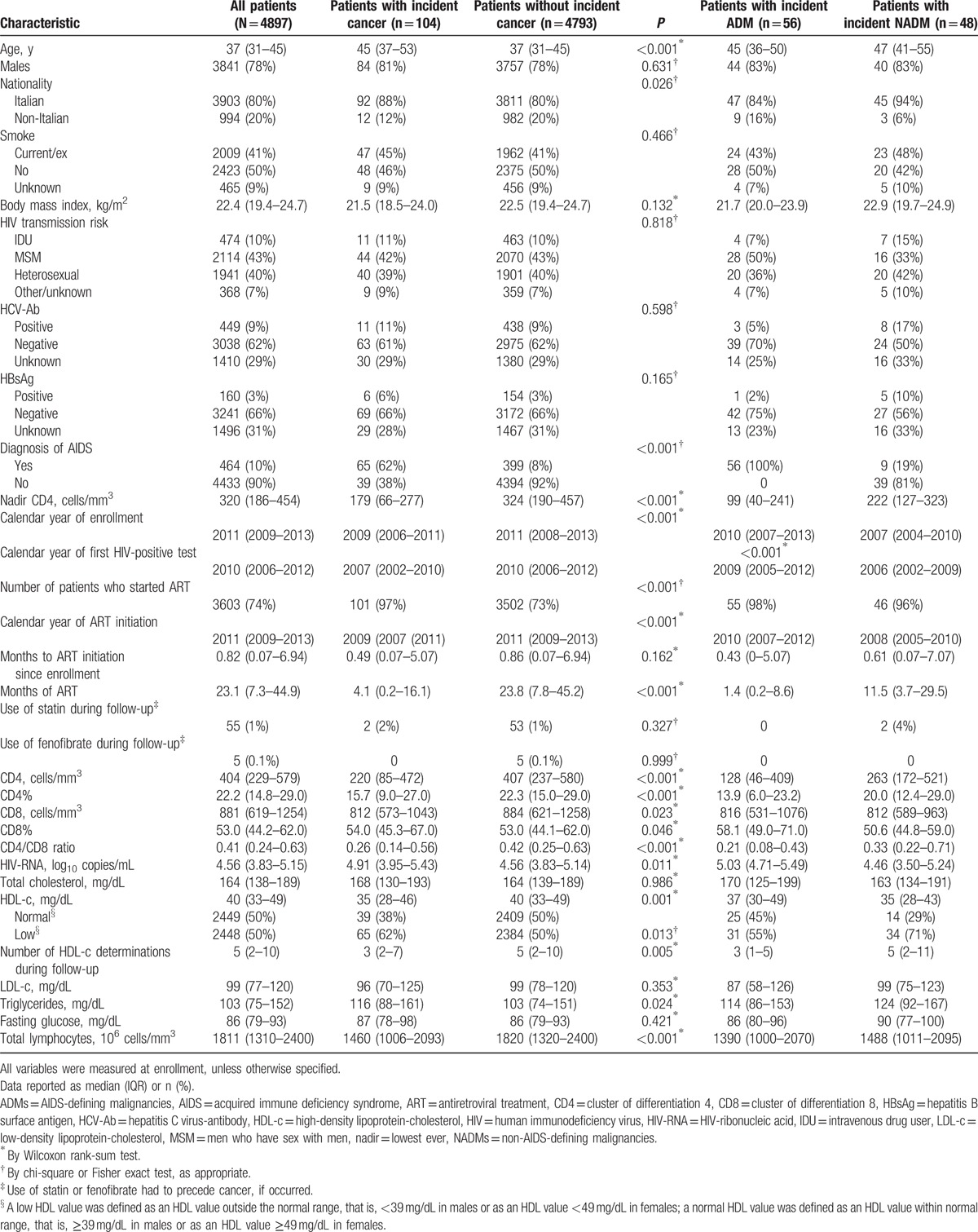
Demographic, clinical, and laboratory characteristics of human immunodeficiency virus-infected patients in the Italian cohort of antiretroviral-naïve patients cohort according to cancer occurrence.

Overall median age was 37 years (31–45), 22% were females, 20% were non-Italian subjects, and 41% were smokers; HIV transmission was mainly by homosexual intercourse (43%). An AIDS diagnosis before enrollment occurred in 10% of patients and CD4+ nadir was 320 (186–454) cells/mm^3^. Very few patients had a positive serologic test for HCV-Ab (9%) and for HBsAg (3%). Median time of follow-up was 1.9 (0.42–3.93) years, accounting for 13,440 PYFU (with incident cancer: 0.96 [0.22–2.29] years; without incident cancer: 1.92 [0.44–3.97] years; *P* = 0.001). During follow-up, 104 diagnoses of cancer were reported (56 ADMs and 48 NADMs); cancer characteristics are depicted in Table 2.

**Table 2:**
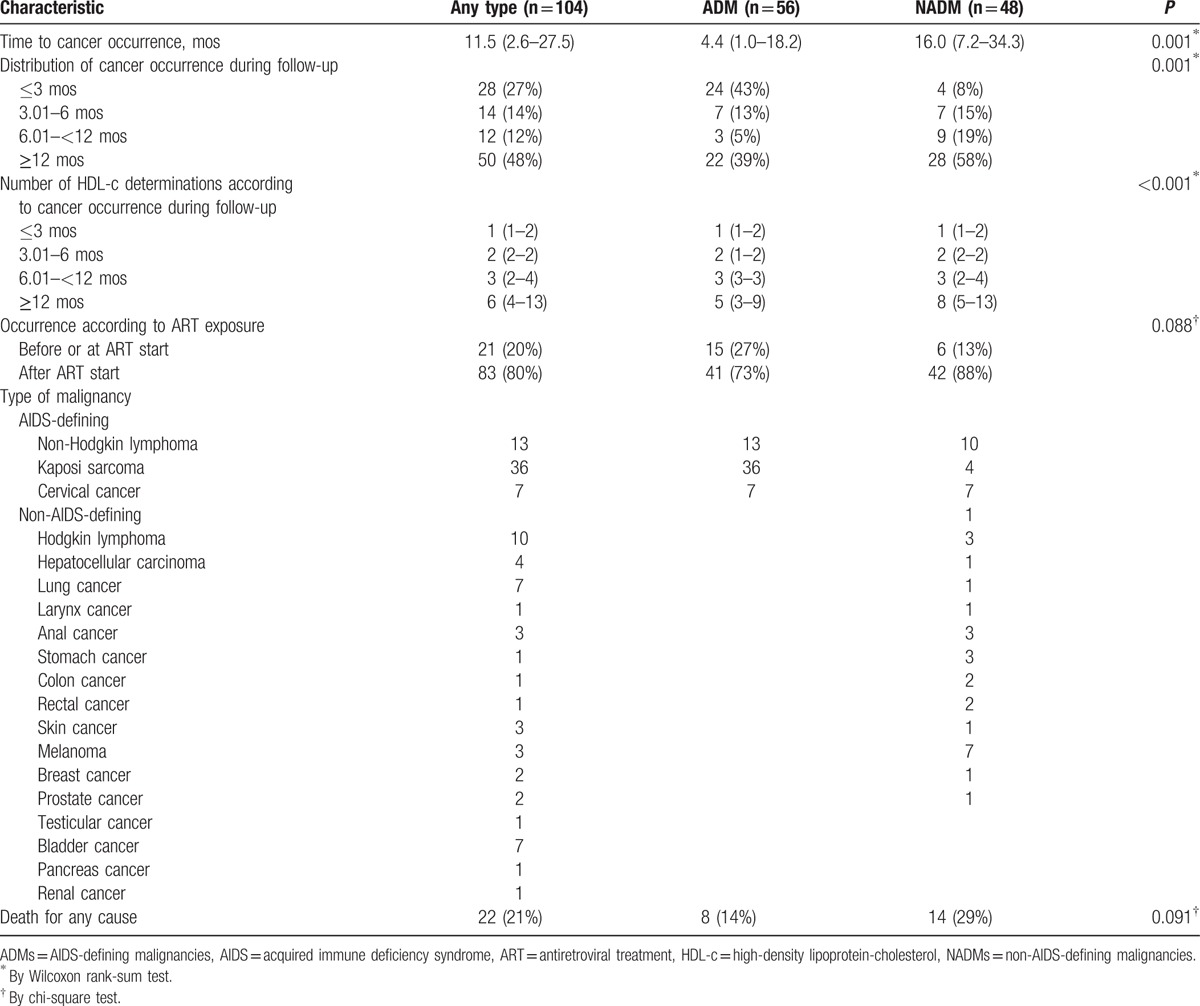
Cancer characteristics (first diagnosis).

Forty-eight per cent of cancers occurred 12 months after enrollment and 80% of cancers were diagnosed after ART initiation. KS (64%) was the most frequent ADM followed by non-Hodgkin lymphoma (23%) and cervical cancer (13%). Hodgkin lymphoma (21%) was the most frequent NADM followed by lung and bladder cancer (for both 15%) and liver cancer (8%).

At enrollment, 2448 (50%) subjects had low HDL-c values, and the median number of the available HDL-c determinations during follow-up was 5 (2–10).

Patients with low as opposed to normal HDL-c values at enrollment were less frequently male (77% vs 80%; *P* = 0.004), more frequently smokers (47% vs 43%; *P* = 0.006), with a previous diagnosis of AIDS (12% vs 7%; *P* < 0.001), started ART during follow-up (79% vs 69%; *P* < 0.001), were older (38 [31–46] vs 36 [30–44] years; *P* < 0.001), with lower CD4 nadir (288 [141–432] vs 348 [235–480] cells/mm^3^; *P* < 0.001), lower CD4+ cell count (351 [164–534] vs 449 [293–618] cells/mm^3^; *P* < 0.001), lower CD4/CD8 ratio (0.35 [0.19–0.57] vs 0·47 [0.30–0.69]; *P* < 0.001), higher HIV-VL (4.74 [4.07–5.36] vs 4.39 [3.67–4.92] log10 copies/mL; *P* < 0.001), lower total lymphocytes count (1765 [1200–2370] vs 1900 [1440–2400] cells/mm^3^; *P* < 0.001), lower total cholesterol (151 [127–177] vs 174 [152–200] mg/dL; *P* < 0.001), lower low-density lipoprotein-cholesterol (LDL-c) (92 [72–114] vs 104 [84–126] mg/dL; *P* < 0.001), and higher triglycerides (124 [88–174] vs 88 [66–124] mg/dL; *P* < 0.001). Patients with low HDL-c values at enrollment also died more frequently during follow-up (2.8% vs 1.5%; *P* = 0.002) as compared with those with normal HDL-c values.

The overall cancer incidence rate was 7.7 (95% CI 6.3–9.2) per 1000 PYFU (ADM: 4.2 [95% CI 3.1–5.3] per 1000 PYFU; NADM: 3.6 [95% CI 2.6–4.6] per 1000 PYFU).

Overall and NADM incidence rates significantly differed between subjects with normal as opposed to those with low values of HDL-c at enrollment (overall: 5.7 [95% CI 4.2–7.8] vs 9.8 [95% CI 7.7–12.6] per 1000 PYFU; *P* = 0.006 by univariate Poisson regression; ADM: 3.7 [95% CI 2.5–5.4] vs 4.7 [95% CI 3.3–6.7] per 1000 PYFU; *P* = 0.353 by univariate Poisson regression; NADM: 2.0 [95% CI 1.2–3.5] vs 5.2 [95% CI 3.7–7.2] per 1000 PYFU; *P* = 0.002 by univariate Poisson regression).

Patients with incident cancer as compared with those without (Table 1) were found to be older (45 [37–53] vs 37 [31–45] years; *P* < 0.001], more frequently with previous diagnosis of AIDS (65 [62%] vs 399 [8%] events; *P* < 0.001), with lower CD4 nadir (179 [66–277] vs 324 [190–457] cells/mm^3^; *P* < 0.001), earlier calendar year of enrollment (2009 [2006–2011] vs 2011 [2008–2013]; *P* < 0.001), lower CD4 cell count (220 [85–472] vs 407 [237–580] cells/mm^3^; *P* < 0.001), lower CD4/CD8 ratio (0.26 [0.14–0.56] vs 0.42 [0.25–0.63]; *P* < 0.001), lower total lymphocytes count (1460 [1006–2093] vs 1820 [1320–2400] cells/mm^3^; *P* < 0.001), higher HIV-VL (4.91 [3.95–5.43] vs 4.56 [3.83–5.14] log10 copies/mL; *P* = 0.011), and lower HDL-c (35 [28–46] vs 40 [33–49] mg/dL; *P* = 0.001) at enrollment.

**Figure 1:**
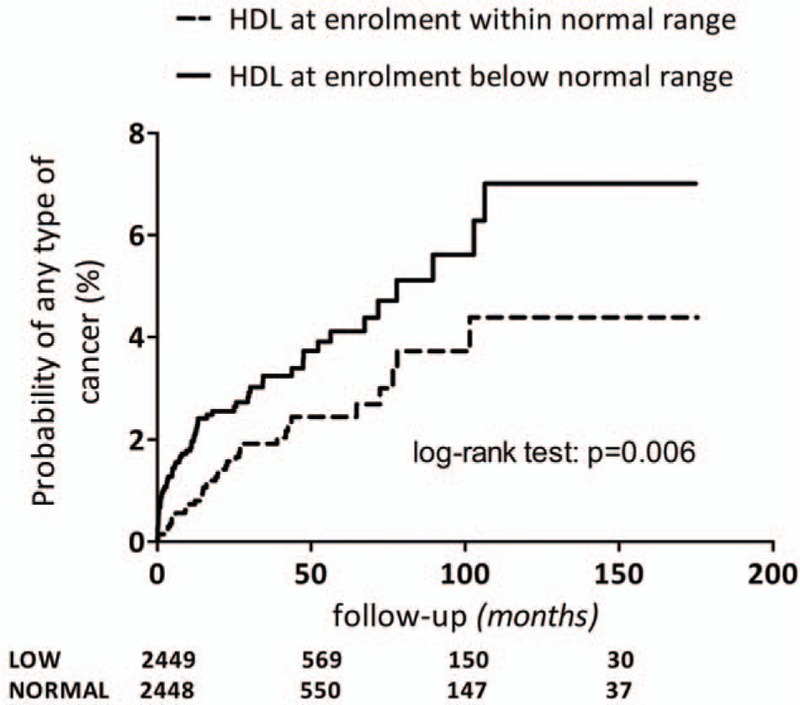
Kaplan–Meier curves estimating the probability of cancer occurrence according to high-density lipoprotein-cholesterol at enrollment below normal range (continuous line) and to normal high-density lipoprotein-cholesterol (dotted line).

Low HDL-c values at enrollment were associated with a higher risk of any type of cancer (crude hazard ratio [HR] 1.72, 95% CI 1.16–2.56, *P* = 0.007) and with a higher risk of NADM (crude HR 2.50, 95% CI 1.35–4.76, *P* = 0.003) as shown in Fig. 1.

At univariate analysis (Table 3), the risk of cancer was associated with older age, not being on ART, low CD4 values, low CD4/CD8 ratio, high HIV-RNA values, low total cholesterol values, low HDL-c values, low LDL-c values, and high values of fasting glucose.

**Table 3:**
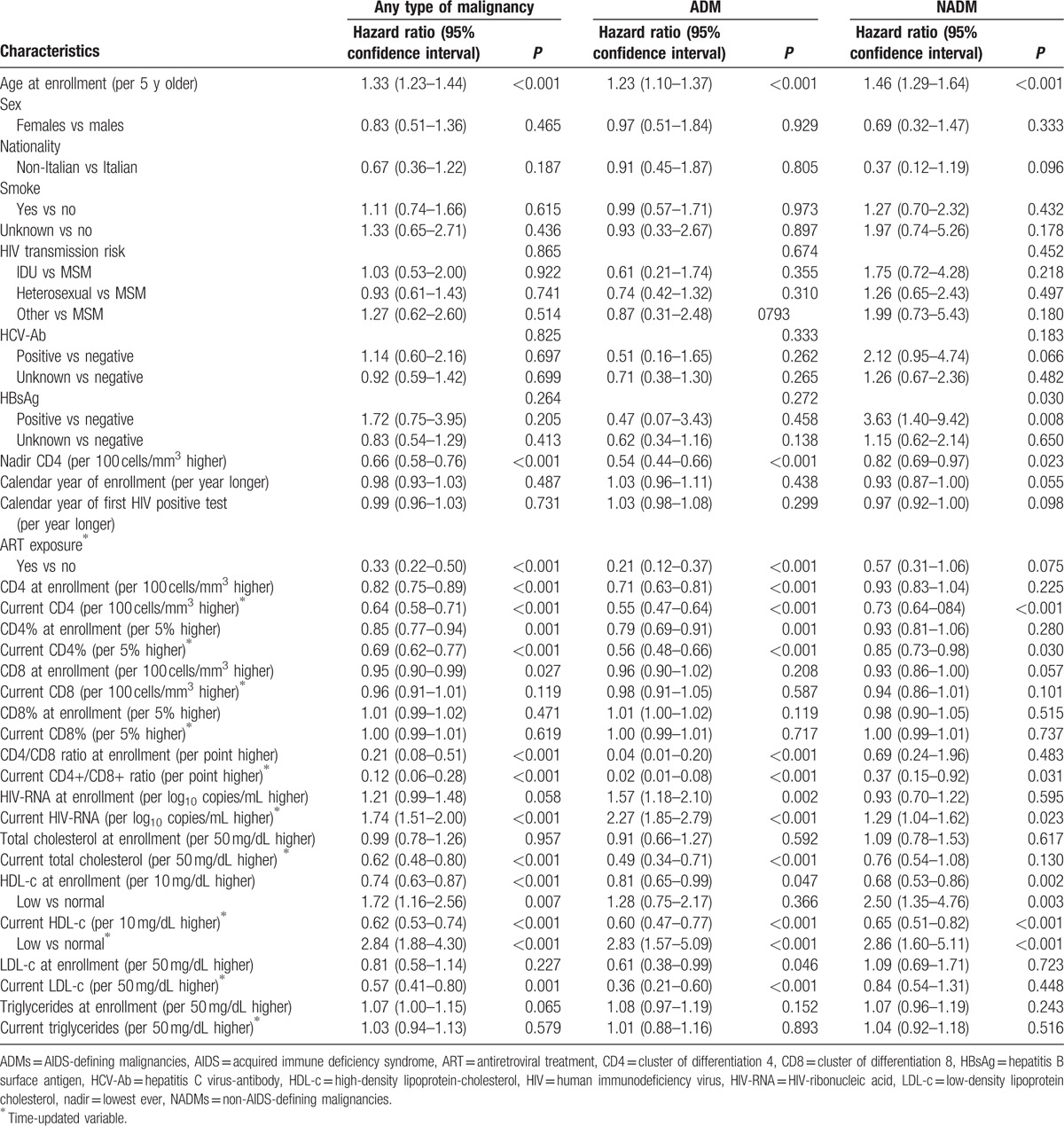
Univariate Cox proportional-hazard models on the risk of any type of malignancy, the risk of AIDS-defining malignancies and the risk of non-AIDS-defining malignancies.

Results of the multivariate analysis are reported in Table 4. Notably, the risk of cancer diagnosis was higher in patients with low current HDL-c values (adjusted HR [AHR] for low vs normal: 1.87, 95% CI 1.18–2.95, *P* = 0.007) and in those with older age (AHR per 5-years older: 1.32, 95% CI 1.20–1.44, *P* < 0.001), low current CD4 levels (AHR per 100 cells/mm^3^ higher: 0.78, 95% CI 0.68–0·91, *P* < 0.001), high current HIV-RNA values (AHR per 1 log10 copies/mL higher: 1.69, 95% CI 1.36–2·11, *P* < 0.001), and more recent calendar year of enrollment (AHR per 1 more recent year: 1.10, 95% CI 1.03–1.17, *P* = 0.005).

**Table 4:**
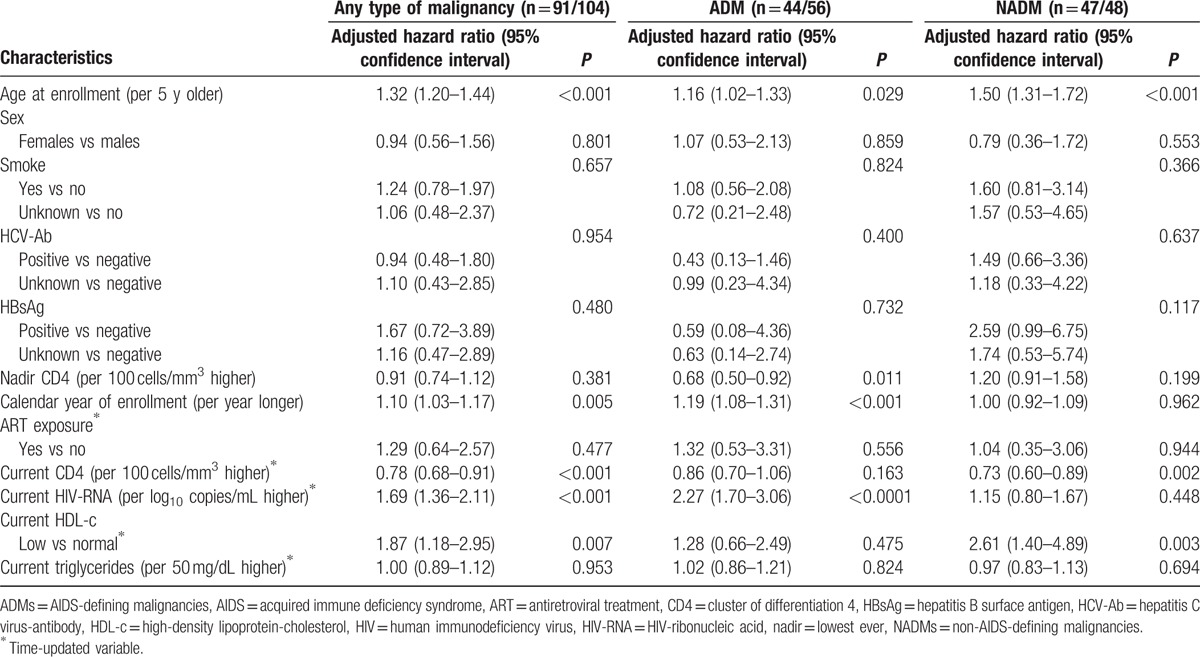
Multivariate Cox proportional-hazard models on the risk of any type of malignancy, the risk of AIDS-defining malignancies, and the risk of non-AIDS-defining malignancies.

Low values of HDL-c still appeared to be a risk factor for risk of ADM (Table 4), although not statistically significant (AHR for low vs normal: 1.28, 95% CI 0.66–2.49, *P* = 0.475); older age (AHR per 5 years older: 1.16, 95% CI 1.02–1.33, *P* = 0.029), low nadir CD4 levels (AHR per 100 cells/mm^3^ higher: 0.68, 95% CI 0.50–0.92, *P* = 0.011), high current HIV-RNA values (AHR per 1 log10 copies/mL higher: 2.27, 95% CI 1.70–3.06, *P* < 0.001), and more recent calendar year of enrollment (AHR per 1 more recent year: 1.19, 95% CI 1.08–1.31, *P* < 0.001) were associated with a higher risk of ADM.

The multivariate model on risk of NADM confirmed associations with low values of HDL-c (AHR for low vs normal: 2.61, 95%CI 1.40–4.89, *P* = 0.003), older age (AHR per 5 years older: 1.50, 95% CI 1.31–1.72, *P* < 0.001), and low current CD4 levels (AHR per 100 cells/mm^3^ higher: 0.73, 95% CI 0.60–0.89, *P* = 0.002).

Additional adjustment for current CD4/CD8 ratio (see Table 5) led to similar conclusions with regard to the effect of low HDL-c values (AHR of any cancer for low vs normal: 1.87, 95% CI 1.14–3.06, *P* = 0.013; AHR of ADM for low vs normal: 1.19, 95% CI 0.56–2.52, *P* = 0.648; AHR of NADM for low vs normal: 2.65, 95% CI 1.38–5.08, *P* = 0.003] with a weak association with CD4/CD8 ratio.

**Table 5:**
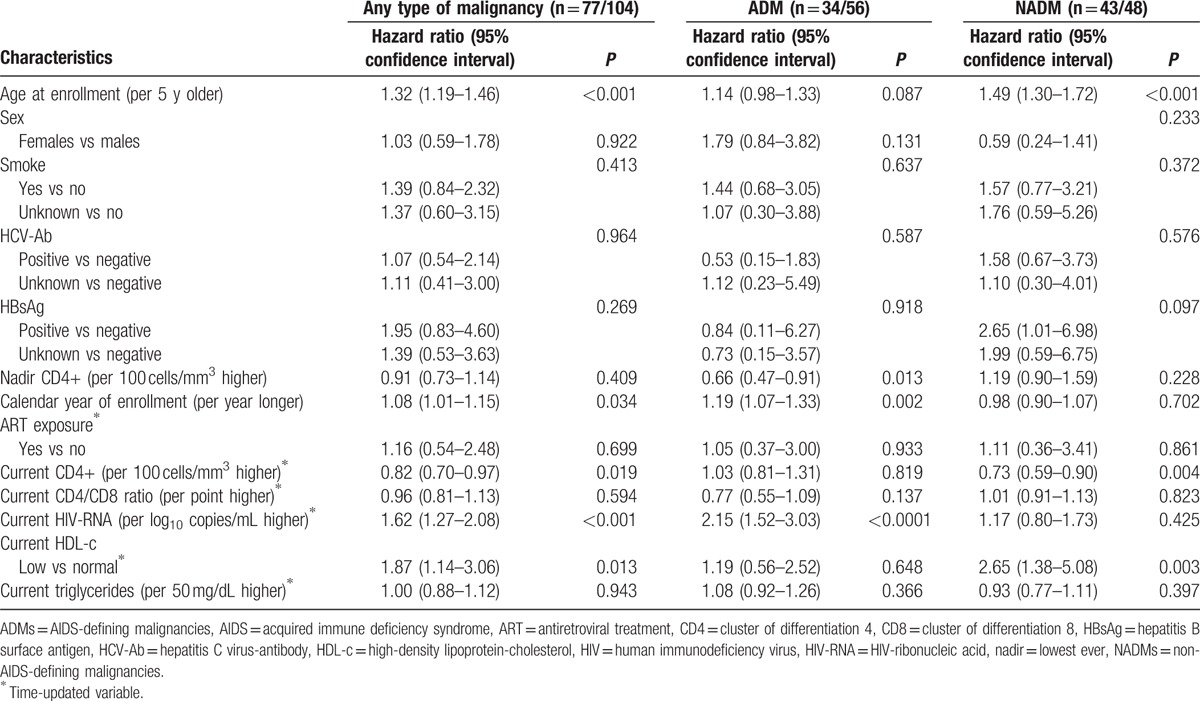
Multivariate Cox proportional-hazard models on the risk of any type of malignancy, the risk of AIDS-defining malignancies, and the risk of non-AIDS-defining malignancies (including CD4/CD8 ratio).

Among patients who had received ART (see Table 6), the independent association between HDL-c and post-ART cancer occurrence of any type or NADM was confirmed (AHR of any cancer for low vs normal: 2.39, 95% CI 1.44–3.96, *P* < 0.001; AHR of ADM for low vs normal: 1.62, 95% CI 0.75–3.48, *P* = 0.221; AHR of NADM for low vs normal: 3.14, 95% CI 1.59–6.21, *P* = 0.001); among the other factors evaluated in the model, the protective effect of a longer exposure to ART on the risk of cancer became also evident in all the 3 models (AHR of any cancer per 1 month longer: 0.94, 95% CI 0.93–0.95, *P* < 0.001; AHR of ADM per 1 month longer: 0.94, 95% CI 0.92–0.95, *P* < 0. 001; AHR of NADM per 1 month longer: 0.94, 95% CI 0.93–0.96, *P* < 0.001).

**Table 6:**
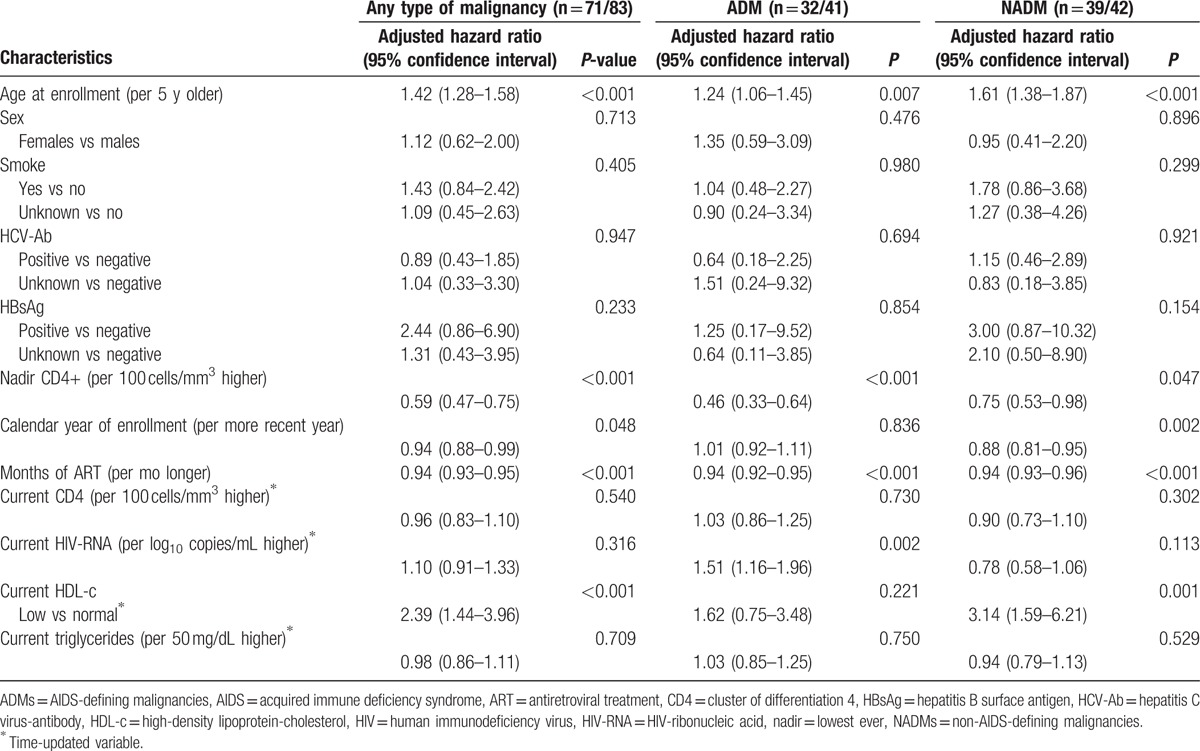
Multivariate Cox proportional-hazard models on the risk of any type of malignancy, the risk of AIDS-defining malignancies, and the risk of non-AIDS-defining malignancies in subjects exposed to antiretroviral treatment.

Finally, we refit the multivariate models after exclusion of cancer diagnoses occurred within the first 6 months since enrollment: current HDL-c remained an independent predictor of any type of cancer or NADM (AHR of any cancer for low vs normal: 1.82, 95% CI: 1.05–3.17, *P* = 0.033; AHR of ADM for low vs normal: 1.15, 95% CI 0.47–2.81, *P* = 0.753; AHR of NADM for low vs normal: 2.51, 95% CI 1.23–5.11, *P* = 0.012).

## 4 Discussion

In a large cohort of ART-naïve patients seen for care in Italy, our primary goal was to evaluate the role of HDL-c levels as a risk factor for cancer, both ADM and NADM.

In our study, we found low HDL-c (<39 for men and < 49 for women) to be an independent risk factor for cancer in HIV, especially for NADM. Indeed, HIV-infected patients with low HDL-c are at 87% more risk of developing malignancies, and at more than double the risk of developing NADMs.

The HDL-c possesses multiple anti-inflammatory properties, such as inhibition of chemoattractant molecules and reduction of expression of adhesion molecules.^[19]^

Inflammation has been purposed as a hallmark of cancer^[20]^ because of activation of various types of gene mutations, chromosomal rearrangement or amplification and inactivation of tumor-suppressive genes, and of infections and inflammatory state by itself.^[21]^

Apolipoprotein A1, the most important proteic component of HDL-c, in vitro has a direct suppressive effect on tumor cells, and in vivo prompts tumor-infiltrating macrophages towards tumor rejection.^[12]^

Moreover, ApoA1-mimetic peptides have demonstrated antitumoral properties in ovarian and colon cancer experimental models in vivo.^[13,14]^

The HDL-c and ApoA1 levels were shown to be inversely correlated with HIV-VL,^[16]^ and they could contribute to the documented increased prevalence of cancer in the HIV population.^[1,2]^

Moreover, recent findings reported that in HIV infection, HDL-c could be dysfunctional in a model in vitro where HDL-c particles extracted from HIV-infected blood samples showed poorest anti-inflammatory activity on preadipocytes.^[22]^

The overall cancer incidence rate in our study was almost double than that of the Italian general population,^[23,24]^ and it was similar to that reported in a recent study of HIV-infected patients in France and in other European countries.^[1,25–27]^ Among traditional risk factors for cancer that may likely explain the increase in incidence rate, our analysis first of all confirmed the role of immune depression, already reported in previous studies.^[1,28–31]^

At multivariate analysis, lower CD4 nadir was a risk factor for ADM, whereas current CD4 cell count was associated with NADM, confirming the role of prior severe immune depression on ADM and suggesting a significant protective role of current CD4 in NADM. At the same time, HIV-VL was associated only with ADM, as expected. The fact that 30% of ADM occurred before ART initiation and 56% of ADM occurred during the first 6 months of follow-up, may likely explain the limited role of CD4 and ART in this subset of patients. And yet, among ADM diagnoses, there was a high proportion of KS diagnoses (64%) that occurred also in patients with high CD4 cell count,^[24]^ and that may explain the lack of association between current CD4 and ADM in our study. Another potential explanation of the limited role of current CD4 in ADM occurrence is that the mean CD4 cell count increase during follow-up was not statistically significant as opposed to what occurred among subjects with a NADM diagnosis (data not shown). The prominent role of CD4 nadir as risk factor for ADM also emerged in the analysis considering only cancers that occurred after ART initiation (Table 6). The strong relationship between lower nadir CD4 cell count and increased ADM risk is well-established.^[29–32]^

As for NADM, there is mounting evidence for an inverse relationship between current CD4 cell count and NADM risk,^[33–36]^and almost previous studies consistently suggest that the current/latest CD4 cell count, reflecting subclinical immunodeficiency, is an important marker of short-term NADM risk (especially infection-related cancers) even in those individuals within high CD4 cell count strata more than 200 to 350/μL.^[37]^ However, nadir CD4 was also independently associated with incident NADM,^[32]^ and also the CD4 cell recovery, which also seemed to be an important factor for controlling the excess risk of some cancers.^[1]^

Another concern is that the lack of association with ART exposure could be due to the high incidence of ADM in the first 6 months of therapy and to the small period of observation. However, we demonstrated a protective role of ART when only cancer occurrence after ART initiation was considered (Table 6).

Among NADM, Hodgkin lymphoma (21%) was the most commonly occurring cancer, followed by lung cancer (15%) and anal cancer (6%), as has been described in the literature.^[1,38]^ We reported 4 HCCs and no association with HCV-Ab positivity, but a significant HR at multivariate analysis associated with HBsAg positivity (HR 2.65, 95% CI 1.01–6.98) (see Table 5). Surprisingly, we found 7 diagnoses of bladder cancer, which is infrequently reported in HIV population.^[39]^ Human papillomavirus (HPV) colonization, predominant in HIV-infected patients,^[40]^ may be responsible for this increased incidence, as demonstrated in a recent meta-analysis.^[41]^

In comparison with the association between low HDL-c and type of cancer in the general population, we found a different pattern of malignancy in HIV-infected patients; breast cancer, endometrial cancer, pancreatic, prostate, and colon and rectal cancer, all associated with low HDL-c in HIV-negative population,^[10,11]^ were poorly represented in our analysis of NADM.

It is possible that immune perturbation due to HIV infection and certain coinfections, such as HPV, HCV, hepatitis B virus (HBV), and Herpes viruses, deeply influence the prevalence of some types of cancer.

We did not find an association with smoking and cancer occurrence. This result could be due to the low incidence of cancers highly associated with smoking occurrence in our study^[42]^: overall, we observed 13 cancers (12.5%) highly associated with tobacco smoking (7 lung, 1 larynx, 1 stomach, and 3 anal cancers). Most of the cancers we observed were, in fact, KS (36 cases, 34.6%), a cancer in which the role of smoking remains to be elucidated.^[43]^

Our study does have some limitations. First, the number of events was rather small, given the brief follow-up, This is particularly true when results are split up into the 2 subgroups of ADM and NADM, since most of the cancer diagnoses (especially ADM) occurred within the first 12 months. We therefore cannot exclude that these diagnoses were already present at HIV infection and that they might be prevalent rather than incident events. As almost one-third of cancer diagnoses occurred within the first 3 months (43% for ADM), only 1 laboratory determination (including HDL-c) was available before cancer diagnosis: for this reason, the benefit provided by the use of time-update covariates in the analysis was fairly limited.

Additionally, our findings could be perceived to be generalized to subjects with recent HIV diagnosis, and those with regular monitoring of HDL-c, and therefore not necessarily representative of all HIV-infected people.

In summary, our study, for the first time, reports an association between HDL-c and risk of cancer in HIV infection. HDL-c is a simple and easy marker that can be performed in every laboratory and could sort patients at higher risk for ADM and especially for NADM. Further follow-up will be needed to confirm our hypothesis.

## Acknowledgments

ICONA Study Group: Board of Directors—A. d’Arminio Monforte (Vice President), M. Andreoni, G. Angarano, A. Antinori, F. Castelli, R. Cauda, G. Di Perri, M. Galli, R. Iardino, G. Ippolito, A. Lazzarin, C.F. Perno, F. von Schloesser, P. Viale; Scientific Secretary—A. d’Arminio Monforte, A. Antinori, A. Castagna, F. Ceccherini-Silberstein, A. Cozzi-Lepri, E. Girardi, S. Lo Caputo, C. Mussini, M. Puoti; Steering Committee—M. Andreoni, A. Ammassari, A. Antinori, C. Balotta, P. Bonfanti, S. Bonora, M. Borderi, M.R. Capobianchi, A. Castagna, F. Ceccherini-Silberstein, A. Cingolani, P. Cinque, A. Cozzi-Lepri, A. d’Arminio Monforte, A. De Luca, A. Di Biagio, E. Girardi, N. Gianotti, A. Gori, G. Guaraldi, G. Lapadula, M. Lichtner, S. Lo Caputo, G. Madeddu, F. Maggiolo, G. Marchetti, S. Marcotullio, L. Monno, C. Mussini, M. Puoti, E. Quiros Roldan, S. Rusconi, A. Saracino; Statistical and Monitoring Team—A. Cozzi-Lepri, I. Fanti, L. Galli, P. Lorenzini, A. Rodano, M. Shanyinde, A. Tavelli; Participating Physicians and Centers—

Italy: A. Giacometti, A. Costantini, C. Valeriani (Ancona); G. Angarano, L. Monno, C. Santoro (Bari); F. Maggiolo, C. Suardi (Bergamo); P. Viale, E. Vanino, G. Verucchi (Bologna); F. Castelli, E. Quiros Roldan, C. Minardi (Brescia); T. Quirino, C. Abeli (Busto Arsizio); P.E. Manconi, P. Piano (Cagliari); J. Vecchiet, K. Falasca (Chieti); L. Sighinolfi, D. Segala (Ferrara); F. Mazzotta, S. Lo Caputo (Firenze); G. Cassola, C. Viscoli, A. Alessandrini, R. Piscopo, G. Mazzarello (Genova); C. Mastroianni, V. Belvisi (Latina); P. Bonfanti, I. Caramma (Lecco); A. Chiodera, A.P. Castelli (Macerata); M. Galli, A. Lazzarin, G. Rizzardini, M. Puoti, A. d’Arminio Monforte, A.L. Ridolfo, R. Piolini, A. Castagna, S. Salpietro, L. Carenzi, M.C. Moioli, C. Tincati, G. Marchetti (Milan); C. Mussini, C. Puzzolante (Modena); A. Gori, G. Lapadula (Monza); N. Abrescia, A. Chirianni, G. Borgia, F. Di Martino, L. Maddaloni, I. Gentile, R. Orlando (Napoli); F. Baldelli, D. Francisci (Perugia); G. Parruti, T. Ursini (Pescara); G. Magnani, M.A. Ursitti (Reggio Emilia); R. Cauda, M. Andreoni, A. Antinori, V. Vullo, A. Cingolani, G. Baldin, S. Cicalini, L. Gallo, E. Nicastri, R. Acinapura, M. Capozzi, R. Libertone, S. Savinelli, A. Latini (Roma); M. Cecchetto, F. Viviani (Rovigo); M.S. Mura, G. Madeddu (Sassari); A. De Luca, B. Rossetti (Siena); P. Caramello, G. Di Perri, G.C. Orofino, S. Bonora, M. Sciandra (Torino); M. Bassetti, A. Londero (Udine); G. Pellizzer, V. Manfrin (Vicenza).
